# Direct retrieval bias for general and specific memories for negatively valenced cues in major depression

**DOI:** 10.1002/ijop.12847

**Published:** 2022-04-16

**Authors:** David John Hallford, Noboru Matsumoto

**Affiliations:** ^1^ School of Psychology Deakin University Melbourne Victoria Australia; ^2^ Division of Psychology, Faculty of Arts Shinshu University Nagano Japan

**Keywords:** Depression, Autobiographical memory specificity, Overgeneral memory, Retrieval process, Meta‐cognition

## Abstract

Major depressive disorder (MDD) is associated with reduced specificity in autobiographical memory. It has been argued that this tendency occurs through a failure of effortful generative retrieval, regardless of valence of cue word. However, we propose that in MDD general memories are likely to be recalled via direct retrieval, and direct retrieval is more likely for negatively valenced cues. To provide a preliminary test of this, a large sample with MDD (*N* = 298; *M* age = 47.2) completed the autobiographical memory test and indicated whether retrievals were generative or direct. Categoric and extended memories for negatively valenced cues were more often directly retrieved than generatively retrieved, and more often than direct retrieval for positively valenced cues. In contrast, categoric and extended memories for positively valenced cues were more often generatively retrieved relative to generative retrieval for negatively valenced cues. Relative to non‐clinical samples, direct retrieval for negatively valenced cues was high. Retrieval method and valence may be moderating processes in the type of memories recalled. This preliminary work presents the possibility of an extension of theory on retrieval tendencies in MDD.

Robust evidence shows that individuals with current major depressive disorder (MDD) show the tendency to recall non‐specific autobiographical memories, such as categoric memories that summarise similar events (e.g., I have made a lot of phone calls to friends recently) or extended memories of periods longer than 24 hours (e.g., first year in high school), rather than memories for specific events that occur at particular times and places (e.g., I had a long phone conversation with a friend about the coronavirus while at home last Wednesday) (Liu et al., 2013). The majority of these findings have been provided through use of the AMT (autobiographical memory test; Williams & Broadbent, 1986) in which participants are given cue word prompts and are asked to report an event from the past for each cue word. In the traditional AMT, participants are explicitly instructed to retrieve specific memories, however, modified versions are also used that include instructions requesting the retrieval of memories, but not explicitly those that are specific (Debeer et al., 2009). The tendency to retrieve general memories is associated with a variety of clinical problems such as worsening the course of depression (Hallford, Rusanov, et al., 2021), impairing social problem‐solving skills, and reducing specificity in episodic future thinking (Williams et al., 2007). Although interventions may alleviate these problems, there is a need to further clarify the retrieval process underlying this tendency in MDD to understand the nature of these deficits, and how to better specify interventions.

Two proposals have previously been made regarding this tendency. The first is that the retrieval of general memories results from a truncated generative retrieval. The Self‐Memory System model (Conway & Pleydell‐Pearce, 2000) and Williams et al.'s (2007) overgeneral memory (OGM) model posited that our autobiographical memories have a hierarchical structure with two voluntary retrieval processes when under instruction to retrieve a specific memory: generative retrieval and direct retrieval. In the generative method, retrieval proceeds via a hierarchical structure (i.e., conceptual self, lifetime period, general memory, specific memory) from the abstract layer to the specific layer, with progressively higher cognitive demands (Conway & Pleydell‐Pearce, 2000; Uzer et al., 2012). In contrast, direct retrieval involves immediate retrieval of a memory, and less cognitive effort is needed to access these mental representations. Several previous studies have considered that the cause of general memory retrieval in MDD is that generative retrieval stops at the general memory level. According to this, general memories are typically retrieved due to this truncation process, not through a process of direct retrieval (Eade et al., 2006; Williams et al., 1999). However, some authors have contended that general memories are also retrieved directly (Dalgleish et al., 2008; Schönfeld & Ehlers, 2017) and this may not indicate truncation, but rather just an immediate mental representation of a general memory. In the context of an AMT that does not explicitly instruct participants to retrieve a specific memory, such responses might indicate a “tendency” to retrieve a general memory rather than a “failure” to retrieve a specific memory. Regardless, the distinction of whether memories are retrieved through searching memory or through a direct retrieval would provide an important insight into how memory is recalled in MDD. The second proposal is that OGM occurs regardless of the cue valence. Griffith et al. (2009, 2012) and Takano et al. (2017) have analysed data in the AMT and found that OGM induced by positive and negative cues converged to one factor.

Recently, however, Matsumoto et al. (2020), have shown increased categoric memory for negatively valenced cues via direct retrieval, and increased categoric memory for positively valenced cues via generative retrieval in individuals with dysphoria and remitted from major depression, compared with controls. They also showed that direct retrieval of categoric memory for negatively valenced cues is common in dysphoria relative to direct retrieval for positively valenced cues and generative retrieval for negatively valenced cues. These findings contradict the above two proposals that OGM results from truncated generative retrieval regardless of the cue valence. Instead, they suggest that, for negative cues, general memories might be more likely to be retrieved directly than generatively, and more often directly than positive cues. On the other hand, for positive cues, generative retrieval might stop at the level of general (categoric) memory (as in the truncated search hypothesis) because of low availability of specific memories. This would be consistent with negative biases in memory in depression (LeMoult & Gotlib, 2019), and suggests that the measurement of retrieval method may be an important factor in understanding how OGM occurs, and how cue valence might affect this.

In a related point, several previous studies (e.g., Eade et al., 2006; Williams et al., 1999) have used abstract cues (e.g., happy, sad) and concrete cues (e.g., wedding, funeral) to distinguish between generative and direct retrieval. That is, they assumed that abstract cues tend to elicit generative retrieval and concrete cues tend to elicit direct retrieval. However, direct retrievals can occur even in abstract cues, and generative retrievals in concrete cues (Harris & Berntsen, 2019; Uzer et al., 2012; Uzer & Brown, 2017). Matsumoto et al. (2020) showed the above results using Uzer et al.'s (2012) method in which participants themselves are asked to determine whether each retrieval is generative or direct. While they showed these trends in retrieval method in individuals with dysphoria and previous major depression, it is not yet clear whether similar trends would be observed in individuals with current MDD.

## Aim of the present study

This study was a secondary analysis of data (see below) and aimed to examine whether individuals with current MDD differed in their retrieval method of general or specific memories based on valence of cues using Uzer's (2012) method. We examined categoric and extended types of general memories and specific memories separately, given their relevance to the course of depression (Hallford, Rusanov, et al., 2021). Based on the above rationale and evidence, we expected there would be both direct and generative retrieval methods would be used for general and specific memories among those with MDD.

In terms of specific hypotheses, we expected that for categoric and extended types of general memories, (a) participants would more frequently report direct retrieval for negatively valenced cues, relative to generative retrieval, and; (b) relative to direct retrieval for positively valenced cues. Conversely, for positively valenced cues, we expected that, (c) participants would more frequently report generative retrieval, relative to direct retrieval, and; (d) relative to generative retrieval for negative cues. The examination of specific memories was considered exploratory, since there are inconsistent findings in previous research (Matsumoto et al., 2020).

## METHODS

### Participants

The participants in this study (*N* = 298) provided the relevant data through online questionnaires as part of a baseline survey for a larger, repeated‐measures study (Hallford et al., 2019; Hallford et al. 2021b). The present sample comprises participants that provided adequate baseline data for inclusion in the present study, but did not necessarily provide follow‐up data for inclusion in the repeated‐measures study. These participants were recruited through advertising on social media platforms (e.g., Facebook, Instagram), and various online groups and forums based in Australia. The relevant inclusion criteria for participants were: ≥18 years of age, resident of Australia, currently experiencing a current Major Depressive Episode (MDE) as determined by the electronic‐Psychological Assessment System (e‐PASS; Nguyen et al., 2015; see below) and a score ≥ 10 on the Patient Health Questionnaire‐9 (PHQ‐9; Kroenke et al., 2010) indicating at least moderate depression.

The mean age of the sample was 47.2 (*SD* = 12.7; range 18–71) and comprised 85.2% females, 13.4% males and 1.3% interest or “other.” Regarding the highest education level attained, 0.7% of participants endorsed primary school, 23.2% high school, 36.2% bachelor degrees, 24.8% diplomas/certificates and 15.1% postgraduate degrees.

All procedures performed in studies involving human participants were in accordance with the ethical standards of the Deakin University Research Ethics Committee and with the 1964 Helsinki Declaration and its later amendments or comparable ethical standards.

Informed consent was obtained from all individual adult participants included in the study.

### Materials

*Electronic Psychological Assessment System* (e‐PASS; Nguyen et al., 2015 **)**. The e‐PASS is an online, self‐report clinical assessment system, with 11 items corresponding to a diagnosis of a Major Depressive Episode from the Diagnostic and Statistical Manual of Mental Health Disorders IV‐TR (DSM‐IV‐TR; American Psychological Association, 2000). The bereavement exclusion was removed to update the system to the DSM‐5 criteria (American Psychological Association, 2013). The questions asked about the frequency of MDE symptoms over the last 2 weeks using a scale from 0 (*not at all*) to 4 (*every day*), and one item relating to severity using a 0 (*no interference/distress*) to 8 (*extremely significant interference/distress*). An algorithm is used to score the e‐PASS in concordance with DSM criteria, whereby if participants endorse at least five symptoms as occurring either *more days than not* or *every day*, at least one of which must be a disturbance in mood or anhedonia, and score three or more on the interference/distress question, they are categorised as experiencing an MDE. In initial validation of the e‐PASS, concordance was acceptable with MDE diagnoses made using the structured, face‐to‐face Mini‐International Neuropsychiatric Interview (Sheehan et al., 1998; Nguyen et al., 2015). A further sample of 50 individuals (aged 18–65) completed the e‐PASS online and the MINI over the telephone as part of an unrelated study currently underway. The concordance between diagnosis on the E‐PASS and the MINI was 94% (*n* = 47/50), confirming its criterion validity for the diagnosis of major depressive disorder.

*Patient Health Questionnaire‐9* (*PHQ‐9*; Kroenke et al., 2010 **).** The PHQ‐9 comprises nine self‐report items referring to the Diagnostic and Statistical Manual for Mental Disorders 4th edition text revision criteria for a Major Depressive Episode (American Psychiatric Association, 2000). Participants rate each item in reference to the frequency of the symptoms over the last 2 weeks on a scale of 0 (*not at all*) to 3 (*nearly every day*), and these items are summed to provide a severity score. The PHQ‐9 has shown good psychometric properties for identifying probable depression and assessing the severity of depressive symptoms (Kroenke et al., 2010). The internal reliability was acceptable in the current study (Cronbach's alpha = .82).

*Autobiographical memory test* (Williams & Broadbent, 1986). Participants were asked to provide written personal memories in as much detail as they could to 10 different cue words, five positively valenced (e.g., success, happy, justice, respect) and five negatively valenced (e.g., pain, stress, danger, cold) presented in alternating order. A “minimal instructions” protocol was used, whereby participants were not asked to retrieve any particular type of memory (Debeer et al., 2009). The cue words were matched on their valence, arousal, and frequency of occurrence. No time limit was given for responses. A random sample of 200 responses were coded as specific (occurring within the space of 24 hours), extended (occurring over more than 24 hours), categoric (a repeated, recurring event), semantic associate (personally relevant, but abstracted information without autobiographical content), or omission (erroneous response, or a present or future thought) by the first author and another researcher blinded to condition and not otherwise associated with the study (Cohen's kappa for inter‐rater reliability was = .84). This second researcher coded the remaining responses. Semantic associates and omissions were coded to describe the AMT responses, but not used in hypothesis testing given they are not autobiographical memories. To assess retrieval method for the autobiographical memories, the participants answered the following question before providing the memory for each of the cue words: “Did this memory come immediately to mind?” There were two response options: “Yes, I thought about it straight away,” or “No, I had to think about it, and search for it in my memory.”

### Procedure

The study received ethics approval from the Human Ethics Committee of the first author's University prior to commencing recruitment. Advertisements were posted online along with a link to the online survey that interested individuals could click on and be taken to a plain language statement. Participants with depression that endorsed items on the e‐PASS indicating that they had a current MDE then went on to complete the measures as part of a larger battery of baseline assessments for the trial. Those who did not meet criteria for an MDE did not complete any further measures. Informed consent was presumed by submission of the responses. No compensation was provided.

### Data analysis

SPSS 25.0 was used for all statistical analyses. Descriptive statistics were generated. First, to examine whether retrieval method differed by memory type a 3 (memory type: categoric, extended or specific) × 2 (retrieval method: direct or generative retrieval) within‐subjects repeated measures ANOVA was used. Then, to test whether retrieval method differed by cue valence, a series of 2 (retrieval method: direct or generative retrieval) × 2 (cue valence: positive or negative) within‐subjects repeated measures ANOVA were used for each memory type, with follow‐up paired sample *t*‐tests to test hypotheses (a–d). The large sample size allowed for statistical power to detect small interaction effects using ANOVAs (Cohen's *f* = .16) and paired samples *t*‐tests (Cohen's *d* ≥ .16) assuming power of .80 and alpha of 0.05. We reasoned this was adequate to detect effects, given that previous studies have shown large effects in differences between valence and retrieval methods between dysphoric and previously depressed individuals (Matsumoto et al., 2020).

## RESULTS

The mean score on the PHQ was 17.5 (*SD =* 3.4), indicating the sample were reporting moderately severe symptoms on average. The average number memories retrieved were as follows: specific: *M* = 3.1, *SD* = 2.1, extended: *M* = 2.1, *SD* = 1.5, categoric: *M* = 0.6, *SD* = 0.8, semantic associate: *M* = 3.6, *SD* = 2.4, omission: *M* = 0.3, *SD* = 0.6. Overall, 61.6% of responses were reported as being via direct retrieval, and 38.4% were via generative retrieval.

In the first ANOVA, a main effect was found for retrieval type with more memories retrieved directly (*M* = .24, *SE* = .008) than generatively overall (*M* = .15, *SE* = .007), *F*(1, 297) = 80.9, *p* < .001, partial *η* ^2^ = .21. A significant interaction effect was found, *F*(2, 594) = 12.4, *p* < .001, partial *η* ^2^ = .04, with follow‐up tests indicating more direct than generative retrievals for categoric memories (*d* = 0.27, *p* < .001), extended memories (*d* = 0.26, *p* < .001), specific memories (*d* = 0.42, *p* < .001), and this effect being larger for specific memories (*p* < .05).

**Figure 1:**
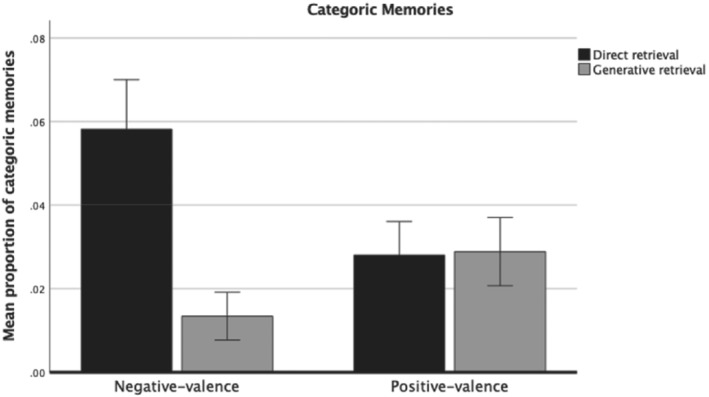
Proportion of categoric memories by retrieval method and cue valence. Error bars: 95% CI.

Focusing on the interaction effects of retrieval method and valence, for categoric memories a significant interaction effect was found, *F*(1, 297) = 29.1, *p* < .001, partial *η* ^2^ = .08 (see Figure 1 for a bar graph of the data). Follow‐up *t*‐tests showed that (a) a higher proportion of categoric memories from negatively valenced cues were directly retrieved compared to generatively retrieved (*M* = .05, *SD* = 0.10 vs. *M* = .01, *SD* = .05; *t*[297] = 6.6, *p* < .001, *d* = .50, 95% CI: 0.34–0.66), and (b) a higher proportion of categoric memories for negatively valenced cues were directly retrieved relative to positively valenced cues (*M* = .05, *SD* = 0.10 vs. *M* = .02, *SD* = .07; *t*[297] = 4.4, *p* < .001, *d* = .34, 95% CI: 0.18–0.50). In contrast, (c) no significant difference was found for retrieval method for positively valenced cues (*M* = .15, *SD* = 0.18 vs. *M* = .13, *SD* = .16; *t*[297] = 0.1, *p* = .884, *d* = .00, 95% CI: −0.16 – 0.16), and (d) a lower proportion of categoric memories were generatively retrieved from negatively valenced cues compared to positively valenced cues (*M* = .01, *SD* = 0.05 vs. *M* = .02, *SD* = .07; *t*[297] = 3.2, *p* < .001, *d* = .24, 95% CI: 0.00–0.32).

**Figure 2:**
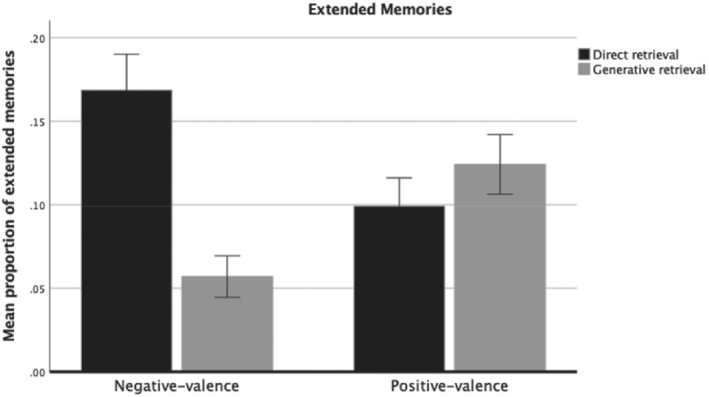
Proportion of extended memories by retrieval method and cue valence. Error bars: 95% CI.

For extended memories, a significant interaction effect between retrieval method and cue valence was found, *F*(1, 297) = 49.3, *p* < .001, partial *η* ^2^ = .14 (see Figure 2 for a bar graph of the data). Follow‐up *t*‐tests showed that (a) a higher proportion of extended memories from negatively valenced cues were directly retrieved compared to generatively retrieved (*M* = .16, *SD* = 0.18 vs. *M* = .05, *SD* = .10; *t*[297] = 8.2, *p* < .001, *d* = .47, 95% CI: 0.35–0.59), and (b) a higher proportion of extended memories for negatively valenced cues were directly retrieved relative to positively valenced cues (*M* = .16, *SD* = 0.18 vs. *M* = .09, *SD* = .14; *t*[297] = 5.4, *p* < .001, *d* = .31, 95% CI: 0.19–0.42). In contrast, (c) no significant difference was found for extended memories from positively valenced cues on the basis of direct or generative retrieval (*M* = .09, *SD* = 0.14 vs. *M* = .12, *SD* = .15; *t*[297] = 1.8, *p* = .070, *d* = .10, 95% CI: −0.21 – 0.01), and (d) a lower proportion of extended memories were generatively retrieved from negatively valenced cues compared to positively valenced cues (*M* = .05, *SD* = 0.10 vs. *M* = .12, *SD* = .15; *t*[297] = 6.1, *p* < .001, *d* = .35, 95% CI: 0.47–0.24).

**Figure 3:**
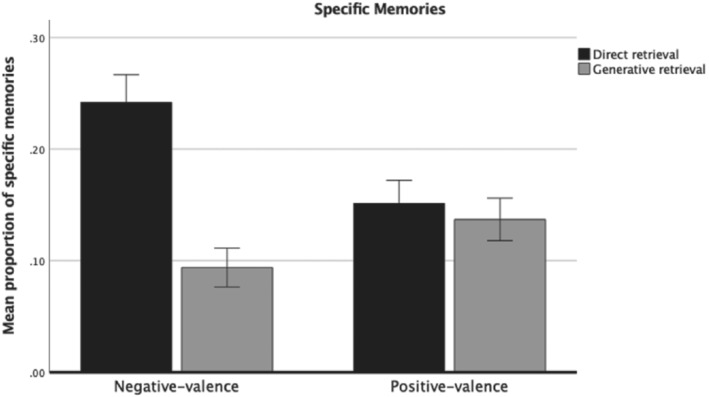
Proportion of specific memories by retrieval method and cue valence. Error bars: 95% CI.

For specific memories, a significant interaction effect between retrieval method and cue valence was found, *F*(1, 297) = 39.9, *p* < .001, partial *η* ^2^ = .11 (see Figure 3 for a bar graph of the data). Follow‐up *t*‐tests showed that (a) a higher proportion of specific memories from negatively valenced cues were directly retrieved compared to generatively retrieved (*M* = .24, *SD* = 0.21 vs. *M* = .09, *SD* = .15; *t*[297] = 9.5, *p* < .001, *d* = .82, 95% CI: 0.65–0.98), and (b) a higher proportion of specific memories for negatively valenced cues were directly retrieved relative to positively valenced cues (*M* = .24, *SD* = 0.21 vs. *M* = .15, *SD* = .18; *t*[297] = 6.1, *p* < .001, *d* = .46, 95% CI: 0.29–0.62). In contrast, (c) no significant difference was found for specific memories from positively valenced cues on the basis of direct or generative retrieval (*M* = .15, *SD* = 0.18 vs. *M* = .13, *SD* = .16; *t*[297] = 0.9, *p* = .339, *d* = .11, 95% CI: 0.04–0.27), and (d) a lower proportion of specific memories were generatively retrieved from negatively valenced cues compared to positively valenced cues (*M* = .09, *SD* = 0.15 vs. *M* = .13, *SD* = .16; *t*[297] = 3.7, *p* < .001, *d* = .25, 95% CI: 0.09–0.41).

## DISCUSSION

The findings indicated that direct retrieval was common among categoric, extended and specific memory types in MDD, and more frequently than generative retrieval particularly in specific memories. As expected, general memories inspired by negatively valenced cues were more likely to be directly retrieved than generatively retrieved, and more likely to be directly retrieved than general memories for positively valenced cues, with approximately moderate‐sized effects. Contrastingly, general memories for positively valenced cues were more likely to involve a generative retrieval process than negatively valenced cues. These results extend on the findings of previous research with individuals with dysphoria and previous major depression (Matsumoto et al., 2020) to individuals with current MDD. Negative categoric and extended memories came more immediately to mind, suggestive of readily accessible mental representations of negative categories of events of individuals with depression. This direct retrieval of general memories to negative cues may partly explain why OGM is a vulnerability factor for depression (Hallford, Rusanov, et al., 2021). Directly retrieved memories, compared to generatively retrieved, have been shown to be more personally significant and emotionally intense. Therefore, when they are brought to might they may negatively bias beliefs or judgements regarding past and future circumstances. This might also be a gateway to rumination, or perhaps a product of it, and therefore a precipitant and maintaining factor in depression (Liu et al., 2017; Nolen‐Hoeksema, 2000). These findings suggest it is possible that when a general memory is retrieved as part of an AMT that explicitly asks for a specific memory, this may reflect direct retrieval of categoric memory and not just failure of a search process.

Another important finding of the present study is that specific memories for negatively valenced cues were also more often judged to be directly retrieval. Previous studies suggest that personal cues attract more direct retrieval (Harris & Berntsen, 2019; Uzer & Brown, 2017). For individuals with current MDD, negatively valenced cues may have resulted in more direct retrieval because of their high self‐descriptiveness nature (Dozois & Rnic, 2015). In comparison, positively valenced cues may have had a lower direct retrieval due to their low self‐descriptiveness. Interestingly, categoric memory and specific memory showed similar patterns for the valence of cue words and in the retrieval process (Figures 1 and 2). It has been suggested that the accessibility or availability of categoric memory and specific memory are linked together (Hitchcock et al., 2019; Matsumoto & Mochizuki, 2019) and share a common neural basis (Renoult et al., 2016). Thus, emotional cues in this study may equally affect both categoric memory and specific memory recall and their retrieval process.

These results may not be consistent with the theory that OGM occurs independently of emotional valence (Griffith et al., 2009; Takano et al., 2017). Among individuals with current MDD, for negatively valenced cues, categoric and specific memories were more likely to be available and both more likely to be judged as directly retrieved. On the other hand, in positively valenced cues, individuals with current MDD may have less available categoric and specific memory, thus, they have to be generatively retrieved with more effort. OGM occurs in both positively and negatively valenced cues in individuals with current MDD, but the retrieval process may be quite different.

Alternative or additional explanation for these results is that there may be a bias in individuals with current MDD to judge their retrieval as direct, which is specific to the reliance on the subjective judgement of retrieval process. Previous studies showed that there are large individual differences in the reaction time of memories judged to be direct or generative retrieval (Harris et al., 2015; Harris & Berntsen, 2019). Furthermore, they also argued that both direct and generative retrieval are reconstructive processes, and an explanation has been put forward that the differences between the two is the subjective effort spent on retrieval. It is also known that individuals with depression have an abnormality of metacognition and confidence in their internal thoughts (Wells, 2009). One hypothesis that can be drawn from this is that individuals with current MDD lack *the ease of retrieval judgement at post‐retrieval* (i.e., meta‐memory process), and are more likely to judge their retrieval for negative cues as direct retrieval in particular. As evidence, regardless of memory specificity, the overall direct retrieval rate for negative cues (74%) is higher compared to previous studies those used emotional cues with traditional instructions for students (35% in Harris & Berntsen, 2019; 48% in Harris et al., 2015; 43–53% in Uzer et al., 2012), and negatively valenced cues with minimal instructions for control groups (Matsumoto et al., 2020; 44% in Study 1, 55% in Study 2). This hypothesis needs to be carefully examined in the future because reaction times were not measured in this study nor other variables that might be related to the subjective judgements. In any case, the phenomenon of memories popping into mind via direct retrieval and involuntary recall is itself a subjectivity‐dependent construct. Rather than questioning the validity of Uzer et al.'s (2012) method of measuring subjective judgements of whether retrieval is generative or direct, we should recognise that the direct retrieval observed in this way is an important construct with implications for understanding and treatment of psychiatric disorders who suffering from direct and/or involuntary retrieval. Future research may investigate whether this bias for direct retrieval for negatively valenced cues might underpin how OGM predicts and maintain depression, and whether this might also be a mechanism of change in memory specificity interventions. Examining retrieval methods in future thinking would also improve understanding of how overgeneral episodic future thinking is a factor in depressive psychopathology, and in other disorders where such deficits are observed (Hallford et al., 2018).

### Further limitation and future directions

While comments can be made regarding differences within the sample with an MDE, between‐group differences using a control group is an important next step. The influence of different AMT instructions also cannot be considered. In traditional instruction (Williams & Broadbent, 1986), participants tend to try generative retrieval because it strongly prompts the retrieval of a specific memory, while the AMT minimal instruction (Debeer et al., 2009) used in this study tends to reflect direct retrieval because participants are asked to report any event. Nevertheless, the direct retrieval rate for negatively valenced cue in this study was relatively high compared with studies using traditional instruction in healthy populations (Harris et al., 2015; Harris & Berntsen, 2019; Uzer et al., 2012; Uzer & Brown, 2017) and minimal instruction in healthy controls (Matsumoto et al., 2020). Furthermore, there can be variation in patterns of responding across different retrieval contexts for the AMT, such as in‐person verbal reporting or non‐computerised handwritten responses (Bunnell et al., 2020). This may also influence the findings and could be investigated beyond the context of this study and those of in‐person computerised responses reported in Matsumoto et al. (2020). Lastly, a proportion of the participants in this study did not complete subsequent time‐points in the larger repeated‐measures study from which this data was drawn. Therefore, the sample may be biased in some way.

## CONCLUSION

The present study showed that direct retrieval of categoric and specific memories for negatively valenced cues is more common, relative to generative retrieval, in individuals with current MDD. In addition, generative retrieval of categoric and specific memories for positively valenced cues is common relative to for negatively valenced cues. Notably, these results are preliminary, but warrant further testing, and suggestive of alternatives to the conventional view that OGM is derived from the attenuation of generative retrieval regardless of the cue valence.
